# Genome-Wide Association Study and Genomic Prediction on Plant Architecture Traits in Sweet Corn and Waxy Corn

**DOI:** 10.3390/plants12020303

**Published:** 2023-01-09

**Authors:** Dongdong Dang, Yuan Guan, Hongjian Zheng, Xuecai Zhang, Ao Zhang, Hui Wang, Yanye Ruan, Li Qin

**Affiliations:** 1 Shenyang City Key Laboratory of Maize Genomic Selection Breeding, College of Bioscience and Biotechnology, Shenyang Agricultural University, Shenyang 110866, China; 2 CIMMYT-China Specialty Maize Research Center, Crop Breeding and Cultivation Research Institute, Shanghai Academy of Agricultural Sciences, Shanghai 201403, China; 3 International Maize and Wheat Improvement Center (CIMMYT), El Batan, Texcoco 56237, Mexico

**Keywords:** genome-wide association study, genomic prediction, plant height, ear height, tassel branch number, sweet corn, waxy corn

## Abstract

Sweet corn and waxy corn has a better taste and higher accumulated nutritional value than regular maize, and is widely planted and popularly consumed throughout the world. Plant height (PH), ear height (EH), and tassel branch number (TBN) are key plant architecture traits, which play an important role in improving grain yield in maize. In this study, a genome-wide association study (GWAS) and genomic prediction analysis were conducted on plant architecture traits of PH, EH, and TBN in a fresh edible maize population consisting of 190 sweet corn inbred lines and 287 waxy corn inbred lines. Phenotypic data from two locations showed high heritability for all three traits, with significant differences observed between sweet corn and waxy corn for both PH and EH. The differences between the three subgroups of sweet corn were not obvious for all three traits. Population structure and PCA analysis results divided the whole population into three subgroups, i.e., sweet corn, waxy corn, and the subgroup mixed with sweet and waxy corn. Analysis of GWAS was conducted with 278,592 SNPs obtained from resequencing data; 184, 45, and 68 significantly associated SNPs were detected for PH, EH, and TBN, respectively. The phenotypic variance explained (PVE) values of these significant SNPs ranged from 3.50% to 7.0%. The results of this study lay the foundation for further understanding the genetic basis of plant architecture traits in sweet corn and waxy corn. Genomic selection (GS) is a new approach for improving quantitative traits in large plant breeding populations that uses whole-genome molecular markers. The marker number and marker quality are essential for the application of GS in maize breeding. GWAS can choose the most related markers with the traits, so it can be used to improve the predictive accuracy of GS.

## 1. Introduction

Maize (*Zea mays* L.) is the most important food, feed, and economic energy crop in the world. Its production safety plays an extremely important role in ensuring national grain production, promoting the development of animal husbandry, and improving people’s quality of life [1,2]. Sweet corn and waxy corn, a new type of fresh edible maize, has been widely planted. It can be used as the replacement of vegetables or fruits, because it tastes sweet and juicy as well as having high nutritional value. The content of vitamins, proteins, lysine, sugar and fat is much higher than that of regular maize [3]. Sweet corn, derived from the mutation in the relative gene regulating the conversion of sugar to starch inside the endosperm of the corn kernel, have a favorable flavor and is planted worldwide [4]. Waxy corn, a variety of maize expressing only amylopectin, has been extensively planted in China and many other countries [5]. Using molecular markers can help to understand the genetic diversity of existing sweet corn and waxy corn germplasm resources and using gene mapping to study plant architecture traits and analyze its genetic basis will help improve the breeding efficiency of sweet corn and waxy corn and further promote the research and development of fresh eating corn varieties.

Ideal plant architecture is critical for increasing plant density. The key components of ideal plant architecture in maize include plant and ear height, leaf angle, ear architecture, root architecture, and tassel architecture. If PH and EH are too high, planting density, lodging resistance, and harvest index will be reduced [6]; If it is too low, it will affect the field permeability, improve the infection rate of diseases and pests and reduce the biological yield [7]. Tassel traits are an important factor affecting yield formation. Overly developed or stunted tassel traits will affect maize yield due to excessive energy consumption, shading, or insufficient pollen supply [8,9]. Considering the continuous population growth, environmental deterioration, and decrease in arable land, moderately increasing planting density is the most effective and simple way to achieve high grain yields. However, higher planting density will promote mutual shading among neighboring plants and limit the efficiency of interception and utilization of light energy of individual plants. The improvement in plant architecture traits during new variety breeding can be used for increasing grain yield with the help of biotechnology. Therefore, the genetic basis for breeding high-yield hybrids needs to be clarified [10]. The PH, EH, and TBN of sweet corn and waxy corn were different from those of regular maize. With the development of molecular marker technology and gene mapping methods, the study of these traits using the genetic mapping method can enhance the role on the genetic basis of these traits, develop molecular markers, and improve the efficiency of breeding.

Genome-wide association studies (GWAS) are powerful tools for gene mapping in plants and animals and have been widely used for genetic analysis of complex quantitative traits in many important crop. In recent years, many scholars have used genome-wide association study (GWAS) to study the loci that control various traits such as PH, EH [11], yield [12], disease resistance [13], and grain dehydration [14] in maize. Yin et al. [15], using the nested association mapping (NAM) population, yielded 264,694 SNPs by genotyping sequencing. A total of 105 SNPs and 22 QTLs were identified by GWAS, which was significantly associated with PH and EH. On chromosome 1, GWAS identified a QTL with high confidence QTL-chr1-ep and performed linkage analysis in two recombinant inbred line (RIL) populations. Wu et al. [16] used genome-wide association analysis and linkage analysis to co-locate the inflorescence size trait, which was measured by panicle main branch number (TBN) and panicle length (TL). A total of 125 QTLs were identified by linkage analysis (63 for TBN and 62 for TL). In addition, 965 quantitative trait nucleotides (QTNs) were identified by GWAS. These QTL/QTNs contain 24 known genes cloned from mutants. In the genetic research of maize traits, scholars generally believe that PH is jointly controlled by major genes and minor polygenes, and the genetic basis is relatively complex, which is a typical quantitative trait inheritance [17]. Therefore, studying maize plant architecture-related traits can not only effectively improve the spatial distribution of maize plants and promote maize growth, but also support for breeding ideal plant architecture and molecular marker-assisted selection (MAS). However, virtually no research has been considered on plant architecture of sweet corn and waxy corn.

Genomic prediction is a method of using markers to predict the genetic value of complex traits in offspring for selection and breeding [18]. When genomic prediction is used for selection, it is called genomic selection (GS). GS is a modified form of marker-assisted selection (MAS) in which the markers from the whole genome are used to estimate the genomic-estimated breeding value (GEBV). A few studies have been conducted to dissect the genetic architecture of plant architecture in maize. In maize, GS has been investigated to improve several major plant architectures, e.g., maize root seedling traits [19], stalk strength [20], root [21], plant height [22], and husk traits [23]. There is no report on the study of plant architecture traits of sweet corn and waxy corn by whole genome selection.

In this study, the association mapping panel comprised sweet corn and waxy corn inbred lines; a total of 477 accessions was used to perform GWAS analysis to dissect the genetic basis of the plant architecture traits of PH, EH, and TBN. The main objectives of the present study are (1) To analyze the genetic diversity of Chinese sweet maize and waxy maize elite inbred lines; (2) Using GWAS to locate and analyze the genetic basis of plant architecture traits, locate the significant SNPs controlling the three traits, identify candidate genes according to GWAS results, and annotate the function of candidate genes; (3) Estimate the prediction accuracy of genome-wide selection. Genetically analyze the maize PH, EH, and TBN by a genome-wide association study, find the quantitative trait loci regulating agronomic traits of maize, and determine a series of candidate genes related to plant growth. The candidate genes and mutation sites that control PH, EH, and TBN were mined, and the genetic evolution rules of key loci were analyzed. It provides theoretical guidance for further developing new germplasm resources and improving varieties more effectively.

## 2. Results

### 2.1. Phenotypic Data Analysis Results

**Figure 1:**
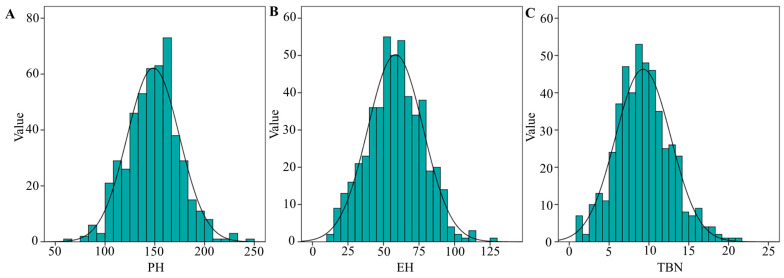
Distribution of phenotypes for PH, EH, and TBN in maize. (**A**) PH. (**B**) EH. (**C**) TBN.

The phenotypic data analysis results of all the target traits of PH, EH and TBN are shown in Table 1. Broad variations were observed for all the three traits in sweet corn and waxy corn. The coefficients of variation (CV) in PH, EH and TBN were 0.17 to 0.23, from 0.33 to 0.36 and from 0.37 to 0.45, respectively. The PH ranged from 63 to 254 cm, the EH ranged from 10–134, and the TBN ranged from 1–26; the absolute values of skewness and kurtosis of PH, EH and TBN were less than 1, indicating a small degree of bias. The frequency distribution of the phenotypes for PH, EH and TBN exhibited approximately near-normal distributions (Figure 1). The heritability for all traits were high and greater than 0.96 in single environment condition. The heritability for PH, EH and TBN in multiple environments analyses were 0.75, 0.79, and 0.72, respectively. Both the genotype and genotype × environment interaction variances were extremely significant (*p* ≤ 0.001) (Table 1).

**Figure 2:**
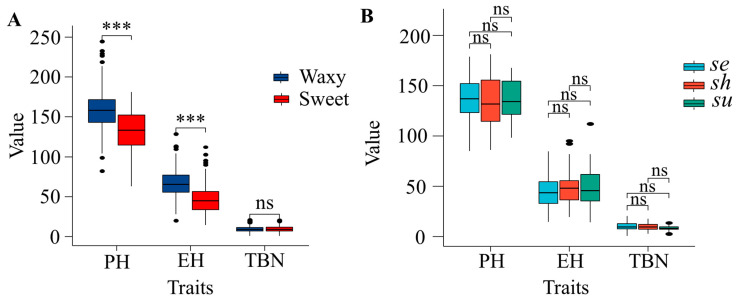
Comparison of PH, EH, and TBN traits among different maize types. (**A**) Comparison between sweet corn and waxy corn. (**B**) Comparison between different genotypes of sweet corn. Asterisk above the box indicates significant differences by Student’s *t* test, *** *p* < 0.001; ns *p* > 0.05.

Between sweet corn and waxy corn, significant difference was observed for PH, as well as for EH. Waxy corn had higher means of PH and EH than that sweet corn (Figure 2A). In the three subgroups of sweet corn, the three plant architecture traits did not show a significant difference (Figure 2B).

**Figure 3:**
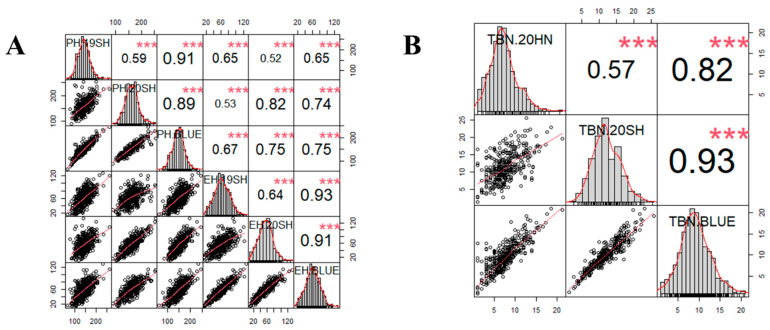
Distributions of and correlations between three relative phenotypic traits. (**A**) Correlation between BLUE and different environments of plant height and ear height. (**B**) Correlation between BLUE and different environments of the number of tassel branches. The frequency distribution histograms of three traits are located on the diagonal line, the area below the diagonal line is the scatter plot of the traits, and the area above is the correlation coefficient between each pair of traits. *** indicate significance at *p* < 0.001.

The results of the correlation analysis between different environments for the same trait and the correlation analysis results between PH and EH were shown in Figure 3A,B. The correlation coefficients between the two environments for PH, EH, and TBN was 0.59, 0.64, and 0.57, respectively. The correlation coefficients of the BLUE values for the same trait between a single environment and multiple environments were high, i.e., greater than 0.80. The correlation coefficients of the BLUE values estimated from multiple environments between PH and EH was 0.75, which were 0.65 and 0.82 in the single environments analysis in 2019 and 2020. The correlations between TBN and other two traits were not estimated.

### 2.2. Results of SNP Characterization, LD Decay Distance, and Population Structure

**Figure 4:**
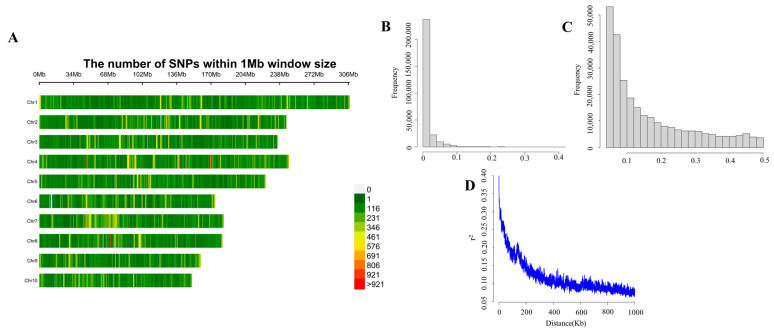
Genotypic diversity and LD decay in the mapping panel. (**A**) Chromosome-specific SNPs density in 1-Mb genomic intervals. The number of SNPs is represented in a green to red scale. (**B**) Frequency distribution of genotypic deletions. (**C**) Distribution of the minimum allele frequency of genotype. (**D**) Whole-genome LD in the entire panel based on 477 maize inbred lines.

The heat map representing the marker density in ten maize chromosomes was showed in Figure 4A. There were 38,013, 32,224, 30,423, 35,688, 28,335, 22,698, 25,987, 24,306, 20,202 and 20,716 SNPS on chromosome 1 to chromosome 10, respectively. The number of markers on chromosome 1 was the most, and the number of markers on chromosome 9 was the least. There were 123.24, 132.24, 127.82, 142.56, 125.18, 125.16, 139.86, 133.255, 123.94 and 135.90 SNPS in 1 per Mb on each chromosome, respectively. The markers were evenly distributed. In the filtered SNP dataset, the average missing rate across the SNPs was 0.12, and the average MAF was 0.16, which was suitable for a subsequent genome-wide association study (Figure 4B,C). We used 278,592 SNPs to evaluate the degree of linkage disequilibrium (LD) attenuation of this association population, which corresponds to 50 kb at *r*^2^ = 0.2 (Figure 4D). LD attenuation was slow, indicating that the higher the degree of domestication, the greater the selection intensity, resulting in a decrease in genetic diversity.

**Figure 5:**
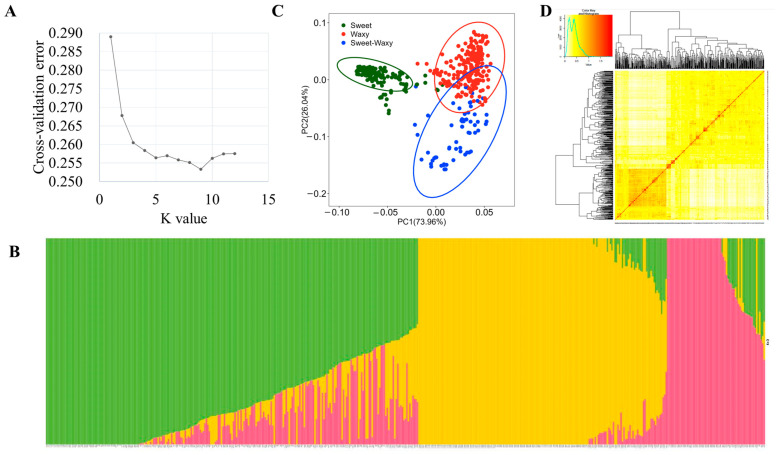
Analysis of genetic diversity. (**A**) ΔK-value of 477 inbred lines. (**B**) The Bayes cluster plot of 477 maize inbred lines when K = 3. (**C**) Principal component analysis. (**D**) Distribution of pairwise relative kinship for 477 maize inbred lines calculated.

Results of the population structure analysis were shown in Figure 5. In general, results of population structure, PCA, and genetic distance or kinship were consistent, and this core collection of waxy and sweet inbred lines could be divided into two or three major groups, according to their pedigrees or genetic backgrounds. When K = 3, the curve slows down, indicating that it was feasible to divide the population into three subgroups (Figure 5A,B). The number of lines in subgroups 1, 2, and 3 was 247, 164, and 66, respectively. The principal component analysis also revealed three subgroups, the first two principal components explained most variances (Figure 5C) corresponding to the three subgroups identified by structure analysis (Figure 5D): sweet corn subgroup, waxy corn subgroup, and sweet–waxy corn mixed subgroup.

### 2.3. Results of GWAS for Plant Architecture Traits

The GWAS was performed by combining the individual location BLUE values of PH, EH, and TBN estimated across environments, the 278,592 high quality SNPs, the first three PCAs, and kinship matrix. A linear mixed model based GWAS was used to control for population structure: both kinship (K) and population structure were taken into account to avoid spurious associations. Q–Q plots showed that the population structure has been well controlled. A mixed linear model (MLM) can reduce the false positive significant markers, but also lead to some false negative significant markers not being identified.

**Figure 6:**
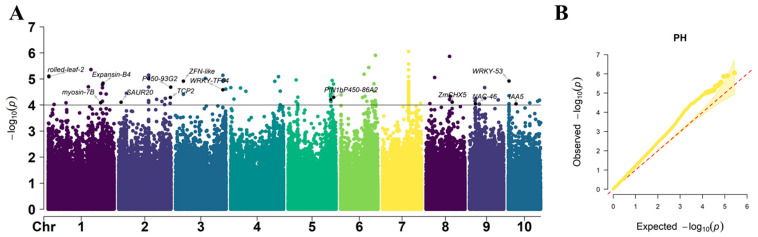
Manhattan plots of GWAS results showing the significant SNPs associated with PH traits. (**A**) Manhattan plot. Each dot represents a SNP. The black solid line represents the threshold of 1× 10^–4^. (**B**) Quantile–Quantile (Q–Q) plots. The red line is the trend line to which the ideal Q–Q plot in each case should correspond.

In total, 184 SNPs significantly (*p* = 1 × 10^−4^) associated with the PH were identified, which were spread across 10 chromosomes (Figure 6). The phenotypic variance explained (PVE) of significant SNPs ranged from 3.5% to 6.4%, with an average value of 4.7%. Out of the total significant SNPs, the maximum number of SNPs were identified on chromosome 7 (85 SNPs) and the minimum number of SNPs were in chromosome 8 (6 SNPs) across locations. The *p*-value of the significantly associated SNPs ranged from 8.8 × 10^−7^ to 9.77 × 10^−5^. The most significant SNPs with the lowest *p*-value were located on chromosome 7, i.e., S7_121735865.

**Figure 7:**
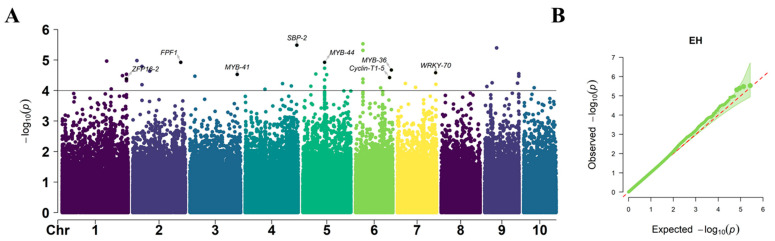
Manhattan plots of GWAS results showing the significant SNPs associated with EH traits. (**A**) Manhattan plot. Each dot represents a SNP. The black solid line represents the threshold of 1× 10^–4^. (**B**) Quantile–Quantile (Q–Q) plots. The red line is the trend line to which the ideal Q–Q plot in each case should correspond.

In total, 45 SNPs significantly (*p* =  1 × 10^–4^) associated with EH were identified, which were located on chromosomes 1, 2, 3, 4, 5, 6, 7, 9, and 10, respectively (Figure 7). The PVE of these significantly associated SNPs ranged from 3.5% to 5.8%, with an average value of 4.4%. Out of these total significant SNPs, the maximum number of SNPs were identified on chromosome 5 (eight SNPs) and the minimum number of SNPs were in chromosome 10, containing only one SNP. The *p*-value of these significantly associated SNPs ranged from 2.94 × 10^−6^ to 9.11 × 10^−5^. The most significantly associated SNP was located on chromosome 6, i.e., S6_34755019. Among the 45 SNPs significantly associated with EH, two were also significantly associated with PH, indicating their pleiotropic effects both on PH and EH. The co-mapping of different traits to the same loci suggested that the genes controlling maize PH and EH have multiple effects.

**Figure 8:**
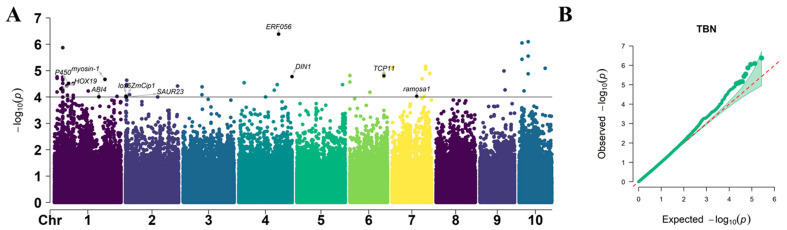
Manhattan plots of GWAS results showing the significant SNPs associated with TBN traits. (**A**) Manhattan plot. Each dot represents a SNP. The black solid line represents the threshold of 1× 10^–4^. (**B**) Quantile–Quantile (Q–Q) plots. The red line is the trend line to which the ideal Q–Q plot in each case should correspond.

In total, 68 SNPs significantly (*p* = 1 × 10^–4^) associated with the TBN were detected, and they were located on chromosomes 1, 2, 3, 4, 5, 6, 7, 9, and 10, respectively (Figure 8). The PVE of these significant SNPs ranged from 3.7% to 7.0%, with an average of 5.0%. Out of all the significantly associated SNP, the maximum number of SNPs were identified on chromosome 1 (25 SNPs) and the minimum number of SNPs were in chromosome 5 (one SNP). The *p*-values of the significantly associated SNPs ranged from 4.11 × 10^−7^ to 9.99 × 10^−5^. The most significantly associated SNP of S4_184008951 was located on chromosome 4. There were no SNPs whose PVE exceeded 10%, indicating that PH, EH, and TBN were traits jointly controlled by a minor gene.

### 2.4. Candidate Genes Revealed by GWAS

Using B73 RefGen_v4 as the reference genome, 483 candidate genes were identified within 50 kb regions either upstream or downstream of the significant SNPs associated with all three plant architecture traits. Table 2 lists the candidate genes with functional annotation on the NCBI website and related to maize growth and development. Based on the expression levels of the candidate genes in plant growth and development, and the functional annotations on the NCBI website, the most promising candidate genes were determined to predict the PH, EH, and TBN in this experiment. Candidate genes were grouped into the following functions: photosynthesis, metabolism, plant hormones, cellular transport, transcriptional regulation, structural proteins, and cell division. These genes can directly or indirectly regulate the growth and development of maize plants. The details of all candidate genes associated with potential SNPs and the functional annotations were presented in Table S1.

### 2.5. Estimation of Genomic Prediction Accuracies

**Figure 9:**
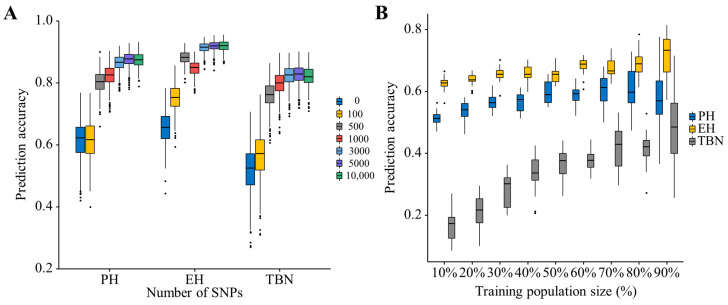
Genomic prediction accuracy of PH, EH, and TBN in the population, (**A**) when the number of SNPs varied from 0 to 10,000. (**B**) when the training population size (TPS) ranged from 10 to 90% of the total population size.

For all three traits of PH, EH and TBN, the prediction accuracies increased rapidly when the number of markers increased from 0 to 500; subsequently, the prediction accuracy increased slightly when the number of markers kept increasing. The differences in prediction accuracies obtained from 3000, 5000, and 10,000 markers were not obvious. It was effective to improve prediction accuracy by adding markers significantly associated with each target trait (Figure 9A).

As the training population size increases, the prediction accuracy gradually improved. When the training population size was 10% of the total markers, the prediction accuracy of PH was 0.51. As the proportion of the training population gradually increased, the prediction accuracy also increased. When the training population size was 80% of the total markers, the prediction accuracy of plant height was evenly distributed around 0.61. When the training population size was 10% of the total markers, the prediction accuracy of EH was evenly distributed at 0.62. With the increasing proportion of training groups, the prediction accuracy also increases. When the training group size was 10% of the total markers, the prediction accuracy of TBN was evenly distributed around 0.16. With the gradual increase of the proportion of training groups, prediction accuracy also increases. When the training group size was 90% of the total markers, the prediction accuracy of TBN was distributed around 0.48. By comparing and analyzing the influence of training population size on the prediction accuracy of the whole genome, the results show that when the training population size increases from 10% to 30% of the total markers, the prediction accuracy increases with the increase of the training population size, and the growth trend was significant. However, when the size of the training group increases from 40% to 80%, the changing trend of prediction accuracy was nearly horizontal. The prediction accuracy of plant height decreased at 90% (Figure 9B).

## 3. Discussion

In the present study, inbred lines representing the core collection of sweet and waxy corn germplasm in China, were used to conduct GWAS and GP analysis on three plant architecture traits, i.e., PH, EH, and TBN. In this study PH, EH, and TBN detected in the association mapping panel also exhibited extensive phenotypic variation and followed a normal distribution. Heritability was at a moderately high level; ANOVA for PH, EH, and TBN showed that the effects of G and G × E interactions were significant, indicating that these three traits were mainly influenced by genetic effects (Table 1). According to the results of GWAS, it was found that the PH, EH, and TBN of maize were typically controlled by multiple genes.

In the analysis of the population structure, although the value at K = 9 was the lowest, when K = 3, the value was obviously slowed down. Coupled with the kinship heat map and PCA analysis in this study, the associated population should be divided into three subgroups, including sweet corn, waxy corn, and sweet–waxy corn (Figure 5). Different populations with the same population type also have great differences in LD decay rate due to their different genetic backgrounds. Domestication selection can lead to a decrease in population genetic diversity and the strengthening of linkage between loci. Therefore, generally, the higher the degree of domestication and the greater the selection intensity of the population, the slower the LD decay rate. Similarly, the decline of population genetic diversity caused by natural selection and genetic drift will also slow down the rate of LD decay [24]. In comparison between LD analysis results and other studies, the value of distance was larger than that in other studies. In tropical maize, the average LD decay distance across all 10 chromosomes was 8.14 kb [25]. In subtropical maize, the average decay distance of the LD across all chromosomes was about 5–10 kb at r^2^ = 0.2 [26]. The smaller the value, the greater the genetic diversity and the greater the genetic relationship between the populations. LD decay rate in this study was similar to that in other sweet corn studies, with the mean length of LD decay decreasing rapidly to 76 kb at a cut-off of r^2^ = 0.2 [27].

In the correlation analysis of phenotypic traits, we found a significant correlation between PH and EH. Many previous studies have also confirmed that PH and EH were related [28]. In addition, GWAS analysis of the three traits found that EH and PH had two overlapping SNPs, which were S3_219824021 and S5_37693709. Therefore, further study on the relevant candidate genes of these loci was helpful to analyze the genetic mechanism of PH and EH in fresh eating maize. Previous research has used QTL mapping and GWAS methods to study the genetic structure of PH and EH traits, but due to the differences in population type and size, marker type and density, and statistical methods used by each research group, the identification of QTL were quite different, and it was difficult for a single study to reveal the genetic structure of maize PH and EH. The previous genome-wide association study of PH and EH was mainly carried out on common maize. This study uses the association group composed of fresh edible maize to overlap the identified significant SNPs and the segments located in the previous study. The SNPs of EH located in this study, S5_101186696, S5_101191399, S5_101191576, S5_101416556, S5_101420833, S5_110982180, S6_117338012 were located in 5.04/05; The SNPs of PH located in this study, S6_109254482, S6_113842238 were located in 6.04/05. These two regions were consistent with the “stable QTL” jointly located by Li using F_2:3_ population and RIL population for PH and EH traits [29]. The SNPs of TBN located in this study, S6_157380718, S6_157381716, and S6_157391371, were located in the QTL and SNPs region of Bins 6.06–6.08 previously identified, indicating that there may be an important region for regulating maize TBN in this region [30,31,32,33]. The results of this study deepen the understanding of the genetic basis of sweet corn and waxy maize plant type traits and contribute to improving the breeding efficiency and breeding new varieties.

Previous studies have cloned some genes that related to TBN, such as mutations in *ramosa1* [34], *ramosa2*, and *ramosa3* [35] with increased TBN numbers. Double mutants of repetitive SBP-box transcription factor genes *unbranched2* and *unbranched3* exhibit a reduced number of tassel branches and an increased number of spike rows [36]. The *ramosa1* gene encodes a putative transcription factor that controls branching architecture in the maize tassel and ear. The candidate gene *Zm00001d020430* mapped by TBN in this study encodes ra1 [37]. The cytochrome P450 (CYP) family plays a key role in plant evolution and metabolic diversification [38]. The genes *Zm00001d017528*, *Zm00001d007924*, and *Zm00001d044120* were cytochrome P450 superfamily proteins, which may regulate the process of plant growth and development and affect the phenotype of plants through the regulation of metabolites in plants. Zinc-finger protein (ZFP) was one of the most important transcription factors in eukaryotes [39,40]. It plays an important role in plant gene expression and regulation, growth, and senescence [41,42]. The candidate genes *Zm00001d022427*, *Zm00001d010380*, *Zm00001d047539*, *Zm00001d034639*, *Zm00001d034642*, *Zm00001d007121*, *Zm00001d038926*, *Zm00001d027312*, *Zm00001d040302*, and *Zm00001d01801* in this experiment encode RING zinc finger domain superfamily proteins and zinc finger CCHC domain proteins, which may regulate the growth and development of plants. Gene *Zm00001d022437*, *Zm00001d044162*, *Zm00001d023332*, *Zm00001d023336*, and *Zm00001d038451* encode a WRKY gene family protein. WRKY were widely involved in regulating rice growth and development by regulating growth regulator-mediated signaling pathways. The plant basic leucine zipper (bZIP) transcription factor protein is encoded by the gene *Zm00001d022442*, *Zm00001d03169* [43]. Glycosyl-phosphatidyl inositol (GPI)-anchored proteins were associated with a variety of growth and developmental mechanisms [44]. The gene *Zm00001d038682* encodes a GPI-anchored protein [45]. These candidate genes may play important roles in plant growth and inflorescence development, but their biological functions require further study. With the development of high-throughput sequencing technology and various gene editing technologies, direct selection of genotypes for crop phenotype improvement has become a reality. This study revealed candidate genes and possible molecular mechanisms regulating PH, EH, and TBN, providing important insights and genetic resources for efficient breeding of maize using genetically improved PH, EH, and TBN.

Genomic selection, especially early selection, was more accurate. Genotyping uses high-density molecular markers to estimate all QTL effects and explain genetic variation for most traits. However, MAS uses fewer markers for trait selection and genomic selection was more accurate than MAS. A previous study shows that GWAS-derived markers improved the prediction accuracy of GS [46]. Consistent with the results of this study, the prediction accuracy gradually increased with the number of significance markers added, and then the increasing trend gradually decreased.

Genomic prediction and GS have been successfully applied to a variety of crops to accelerate genetic gain and improve complex traits in breeding programs [47,48]. The prediction accuracy increases with the increase of the panel TPS, when the TPS increases from 10% to 30%, the prediction accuracy increases rapidly, and when the TPS was further increased, the prediction accuracy increases slightly. If 80% of the total genotypes were used as the training set, the prediction accuracy was higher, and the standard error was smaller. Noman et al. results showed that when the training population was smaller, the prediction accuracy increases as the modeled population increases [49]. However, beyond a certain point, the growth rate of prediction accuracy becomes very low, and breeders can decide on an acceptable prediction accuracy based on the actual situation.

## 4. Materials and Methods

### 4.1. Plant Material

This study utilized an association mapping panel composed of 477 fresh edible maize inbred lines, in which 190 sweet corn inbred lines and 287 waxy corn inbred lines were collected or developed by Shanghai Academy of Agricultural Sciences, China. This panel represents a core collection of sweet corn and waxy corn germplasm in China, and includes most of the parents of the recently released waxy corn and sweet corn varieties. The 190 sweet corn inbred lines could be divided into three subgroups, i.e., enhanced sweet corn, super sweet corn and ordinary sweet corn, according to the sweetness regulatory genes of Sugar-1 (*su1*), shriken-2 (*sh2*) and Sugar Extender (*se*).

### 4.2. Phenotyping and Experimental Design

We evaluated 477 sweet corn and waxy corn inbred lines; and three plant architecture traits of PH, EH, and TBN were measured. The association panel of fresh edible maize was planted at Zhuanghang Experimental Station (N 30°53′, E 121°23′) of the Crop Research Institute of Shanghai Academy of Agricultural Sciences in Shanghai, and at the winter season breeding station (N 18°51′, E 110°03′) in Lingshui County, Hainan Province in 2019 and 2020. The phenotypic data of PH and EH were collected in the summer of 2019 and 2020 from the trials planted in Shanghai, and the phenotypic data of TBN were collected from the trials planted in Hainan in the summer of 2020, and in Shanghai in the winter of 2020. A single row plot was planted with 2.5 m in length and 0.6 m between plots, with 0.25m between plants, and at a planting density of 52,500 plants ha^−1^, A randomized complete block design with two replications per trial was applied. Other field measures were implemented following conventional management practices.

At the maturity stage, after the plant height of the maize inbred line in the natural population was stable, five plants from each row were randomly selected and measured with a tower ruler. The mean value of each trait was used for association analysis. The length from the root to the top of the tassel was the PH of the maize inbred line. EH is measured as the length from the root of the maize to the knot of the uppermost ear of the maize.

### 4.3. Phenotypic Data Analysis

The phenotypic data were analyzed using Microsoft Excel 2007 software to generate descriptive statistics, including the mean, minimum, maximum, standard deviation (SD), coefficient of variation (CV), skewness and kurtosis. The coefficient of variation was calculated as CV (coefficient of variation) = SD (standard deviation)/mean. The frequency distribution of phenotypic data was also checked using Microsoft Excel 2007 software. The kurtosis and skewness were used to estimate the frequency distribution normality. Corrplot in R was used to generate plots using Pearson correlation analysis.

Best Linear Unbiased Estimator (BLUE) and generalized heritability were estimated in META-R [50].

The formula for calculating the BLUE value is:

Y*_ijk_* =*μ* + Rep*_i_*+ Block*_j_*(Rep*_i_*) + Gen*_k_* + *ε_ijk_*

where Y*_ijk_* is the plant architecture trait, *μ* is the overall mean effect, Rep*_i_* is the effect of the *i*th replicate, Block*_j_* (Rep*_i_*) is the effect of the *j*th incomplete block within the *i*th replicate, Gen*_k_* is the effect of the *k*th genotype, and *ε_ijk_* is the effect of the error associated with three factors.

The formula for calculating the generalized heritability is:

$$H^{2}=\frac{{\sigma_{g}}^{2}}{{\sigma_{g}}^{2}+{\sigma_{\mathrm{ge}}}^{2}/n\mathrm{Env}+{\sigma_{\varepsilon }}^{2}/(n\mathrm{Env}\times n\mathrm{Rep})}$$

where σ_g_^2^ and σ_ε_^2^ are the genotype and error variance components, respectively, σ_ge_^2^ is the variance of the G × E cross-variance component, nEnv is the number of environments, and nRep is the number of repetitions. To calculate BLUE and generalized heritability, all effects were declared as random.

### 4.4. Genotyping and Genotypic Data Analysis

For genotyping, fresh young leaves of all accessions were collected, and genomic DNA was extracted using a DNA extraction kit. All samples were sent for genotype detection at Novogene Company using the single nucleotide polymorphism (SNP) Illumina platform. The panel of 477 inbred lines was genotyped on the Illumina platform, and the reference genome was B73 RefGen_v4 for SNP calling. The raw reads were filtered via a standard quality control (QC) process, and the clean reads were obtained for SNP calling. A total of 108,457,756 SNPs were obtained. SNP calling using VCFtools software, the SNPs with missing rate (<20%) and minor allele frequency (MAF > 0.05) were retained, resulting in a final set of 278,592 high-quality SNPs.

### 4.5. Analyses of Linkage Disequilibrium (LD), Population Structure, GWAS, and LD Block Analysis

Population structure analysis: a model-based clustering algorithm in ADMIXTURE Software Version 1.3 [51] was applied. Preliminary analysis was performed in multiple runs by entering consecutive K values from 1 to 12. A five-fold cross-validation procedure was performed for each value of K. The most likely K value was determined using the cross-validation value of ADMIXTURE. Inbred lines with a membership probability greater than 0.5 were assigned to the corresponding clusters and plotted using TBtools software v1.098727 [52]. Principal component analyses (PCA) and clustering analyses were performed in R.

The PopLDdecay 3.40 software (https://github.com/BGI-shenzhen/PopLDdecay (accessed on 13 April 2022)) [53] and perl scripts were used to evaluate linkage disequilibrium (LD) to determine the number of markers required for GWAS, and to determine the detection efficiency and accuracy of GWAS.

The GWAS analysis was conducted in TASSEL 5.0 software [54] by incorporating PCA + K in a mixed linear model. The population structure (PCA) and kinship calculated among individuals were used to adjust the population structure. For the PCA method, the first three PCs (PC1, PC2, and PC3) that were determined from a scree plot constructed from PCs were included in the model as fixed-effect covariates to adjust population stratification. Considering the rigor of the mixed linear model, we conservatively chose −log10 (*p*-value) of 4.0 as the threshold to determine the SNPs significantly associated with the target traits of PH, EH, and TBN, respectively. The Manhattan plot and quantile–quantile (Q–Q) plot were produced using the “CMplot” package in R. The proportion of the explained phenotypic variation by each marker was estimated by the phenotype variance explained. Linkage disequilibrium heat maps were constructed using “LDBlockShow” [55].

### 4.6. Candidate Gene Identification and Annotation

All the putative candidate genes within 50 kb of the detected loci were identified. The expression data and gene annotation information were collected from the maizeGDB database (http://www.maizegdb.org (accessed on 19 May 2022)). The physical locations of the genes and SNPs were based on the maize B73 RefGen_V4 genome. The annotation functions and related information of the candidate genes are obtained from the Maize Genetics and Genomics Database and the National Center for Biotechnology Information (http://www.ncbi.nlm.nih.gov/ (accessed on 1 June 2022)).

### 4.7. Genomic Prediction Analysis

Genomic prediction analysis was conducted with the Ridge Regression Best Linear Unbiased Prediction (RRBLUP) model in R [56]. To estimate the effect of marker density on GP accuracy, the different number of significance markers identified by GWAS—100, 500, 1000, 3000, 5000, and 10,000—were selected to estimate prediction accuracy for all the target traits. At each marker density, SNPs were randomly selected 500 times, and a five-fold cross-validation scheme with 500 repetitions was applied. To explore the effect of training population size on the estimation of the prediction accuracy, training population sizes increasing from 10% to 90% of the total markers, with 10% of the total markers interval, were set to estimate the prediction accuracy for all the target traits.

## Figures and Tables

**Table 1:** Descriptive statistics, variance components, and broad-sense heritability (H^2^) response to PH, EH, TBN in the population.

Trait	Environment	Range	Mean	Skewness	Kurtosis	CV (%)	Variations			H^2^
							G	E	G × E	
PH	20SH	89.00–254.00	160.61 ± 26.92	0.37	0.50	0.17	702.62 **	62.58 **		0.97
	19SH	63.00–264.00	139.79 ± 31.94	−0.85	0.31	0.23	686.65 **	165.79 **		0.93
	BLUE	63.00–244.67	148.53 ± 26.31	0.10	0.50	0.18	447.64 **	118.75 **	254.99 **	0.75
EH	20SH	10.00–134.00	58.14 ± 20.82	0.18	0.10	0.36	414.84 **	48.63 **		0.96
	19SH	14.67–128.67	59.13 ± 20.62	−0.05	0.16	0.35	400.47 **	68.89 **		0.95
	BLUE	14.33–128.67	58.45 ± 19.46	0.12	−0.04	0.33	284.23 **	59.60 **	129.65 **	0.79
TBN	20HN	1.00–21.33	7.32 ± 3.33	0.53	0.77	0.45	9.28 **	2.17 **		0.93
	20SH	1.00–26.00	11.54 ± 4.05	0.34	−0.01	0.35	15.62 **	2.5 **		0.95
	BLUE	1.00–20.83	9.27 ± 3.47	0.32	0.31	0.37	7.31 **	2.34 **	4.97 **	0.72

** correlation is significant at *p* < 0.01.

**Table 2:** Candidate genes for each significant SNP associated with traits and their encoding products.

Trait	Chr	SNP Physical Position	Gene ID	Encoding	Functions
EH	1	298683020	Zm00001d034639	Zinc finger protein ZAT12	transcriptional regulation
			Zm00001d034641	ZFP16-2	other
			Zm00001d034642	Zinc finger protein ZAT11	transcriptional regulation
	2	222818258	Zm00001d007123	FPF1	other
			Zm00001d007121	CW-type Zinc Finger	transcriptional regulation
	3	219824021	Zm00001d044117	MYBR41	transcriptional regulation
			Zm00001d044120	cytochrome P450 CYP51H12	metabolism
			Zm00001d044121	auxin-like 1 protein	plant hormones
	4	240547324	Zm00001d053756	SBP-domain protein2	other
			Zm00001d053753	calmodulin binding protein	metabolism
	6	158598786	Zm00001d038496	Cyclin-T1-5	cell division
	6	167169385	Zm00001d038930	Transcription factor MYB36	transcriptional regulation
	7	177703100	Zm00001d022437	probable WRKY transcription factor 70	transcriptional regulation
			Zm00001d022440	ABI32 ABI3VP1 type transcription factor	plant hormones
			Zm00001d022442	bZIP transcription factor	transcriptional regulation
PH	1	2773087	Zm00001d027317	rolled leaf 2	other
	1	244295536	Zm00001d032945	myosin-7B	structural proteins
	1	255274670	Zm00001d033231	Expansin-B4	other
	2	11369186	Zm00001d002374	SAUR20—auxin-responsive SAUR family member	plant hormones
	2	241314625	Zm00001d007869	UDP-glycosyltransferase 71B1	cellular transport
	2	242857556	Zm00001d007924	cytochrome P450 93G2	metabolism
	3	36650639	Zm00001d040302	Zinc finger CCCH type domain-containing protein ZFN-like 1	transcriptional regulation
	3	220846626	Zm00001d044162	WRKY-TF64	transcriptional regulation
	5	198868106	Zm00001d017528	cytochrome P450 86A2	metabolism
	5	212239163	Zm00001d018016	putative RING zinc finger domain superfamily protein	transcriptional regulation
	8	121528270	Zm00001d010601	ZmCHX5	other
	9	28153529	Zm00001d045600	NAC46	transcriptional regulation
	10	3595365	Zm00001d023332	putative WRKY DNA-binding domain superfamily protein	transcriptional regulation
	10	36382301	Zm00001d024008	auxin-responsive protein IAA5	plant hormones
TBN	1	29829368	Zm00001d028304	Homeobox-leucine zipper protein HOX19	
	1	37949776	Zm00001d028515	NCBP	other
	1	64922019	Zm00001d029289	putative cytochrome P450 superfamily protein metabolism	
	1	204436402	Zm00001d031861	ethylene-responsive transcription factor ABI4	plant hormones
	1	232642160	Zm00001d032637	myosin 1	structural proteins
	2	2113968	Zm00001d001864	uracil-DNA glycosylase	
			Zm00001d001865	ZmCip1, cytokinin-inducible protein	plant hormones
	2	3567341	Zm00001d001961	SAUR23—auxin-responsive SAUR family member	
	2	4131167	Zm00001d001995	ribulose bisphosphate carboxylase/oxygenase activase 2, chloroplastic	photosynthesis
			Zm00001d002000	linoleate 9S-lipoxygenase6	
	4	184008951	Zm00001d052229	ERF056	Other
	4	246401386	Zm00001d054093	senescence-associated protein DIN1	transcriptional regulation
	6	157380718	Zm00001d038444	Transcription factor TCP11	transcriptional regulation
	7	113522699	Zm00001d020430	ra1-ramosa1	Other

## Data Availability

Data available in a publicly accessible repository.

## Supplementary Materials

The following supporting information can be downloaded at: https://www.mdpi.com/article/10.3390/plants12020303/s1, Table S1: SNPs, chromosomal positions and candidate genes identified by GWAS.
